# IDQuAD: Infectious disease question and answering dataset

**DOI:** 10.1371/journal.pone.0333075

**Published:** 2025-10-09

**Authors:** Soonchan Kwon, Sujeong Hur, Beakcheol Jang

**Affiliations:** Graduate School of Information, Yonsei University, Seoul, South Korea; Yarmouk University, JORDAN

## Abstract

While large language models (LLMs) have made significant advances in various fields, The study of applying LLMs to infectious disease-specific tasks has lagged behind. This study addresses this gap by introducing the Infectious Disease Question and Answering Dataset (IDQuAD), which is a novel dataset designed to train and evaluate LLMs in infectious disease-related queries. IDQuAD is constructed using medical papers, patents, and news, and employs innovative methodologies such as generating answers before questions and using counterfactual thinking to enhance the quality of the Question Answering (QA) pairs. In the experimental phase, we fine-tuned the Mistral-7B model on the IDQuAD dataset to test the effectiveness of our proposed datasets on LLM performance in QA tasks related to infectious diseases. The fine-tuned Mistral-7B model demonstrated substantial performance improvements, with its EM score increasing from 28.49% to 65.47% in the one-shot setting. Additionally, we evaluated other LLMs across various setups. Among all models tested, our fine-tuned model achieved the highest performance across metrics and settings. In conclusion, this study introduces IDQuAD as a foundational dataset for infectious disease research, demonstrating the effectiveness of fine-tuning LLMs and paving the way for future advances in dataset development and LLM refinement for infectious disease tasks.

## Introduction

The COVID-19 pandemic has caused severe social and economic damage around the world, highlighting the importance of a rapid and effective response to infectious diseases [1,2]. The rapid spread and complexity of the pandemic have strained existing healthcare systems and epidemic prevention schemes, and efficient technological responses have been and continue to be in demand [3]. Under these circumstances, advances in LLMs, which can process and utilize information without the constraints of time and space, have the potential to play a critical role in the response to infectious diseases.

**Fig 1:**
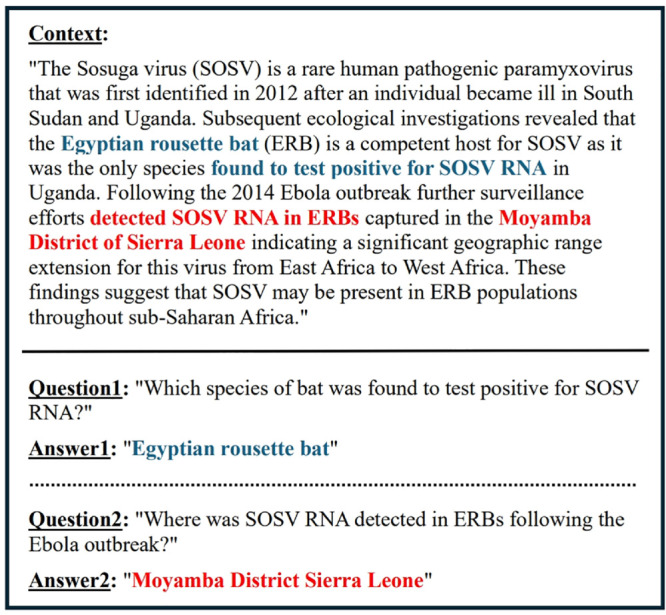
Example of context, question, and answer pair from the IDQuAD dataset.

LLMs have shown excellent performance in solving various problems by learning from large amounts of data, and there has been active research on a variety of domain-specific LLMs and datasets, including medicine, for training and evaluation [4,5]. However, despite the rapid advancements of LLMs in the medical field, research specific to infectious diseases has been relatively slow. The most pressing issue is the significant lack of infectious disease-specific datasets, particularly QA datasets, which are essential for training and evaluating LLMs. This scarcity hinders the development of accurate LLMs for infectious diseases and limits the ability to respond quickly and effectively to outbreaks [6]. To address these challenges, we propose the Infectious Disease Question and Answering Dataset(IDQuAD). An example of the proposed IDQuAD can be found in Fig 1.

The main objective of this study is to create a comprehensive dedicated, and high-quality dataset for infectious diseases that will serve as a QA benchmark. In doing so, this benchmark is designed to enhance the performance of language models in generating accurate and relevant responses to questions related to infectious diseases. Furthermore, this study aims to lay the groundwork for building infectious disease QA datasets that can serve as a practical tool for public health responses in the event of future outbreaks.

- This study introduces IDQuAD, a cutting-edge dataset specifically tailored for queries related to infectious diseases. IDQuAD is expected to serve as a valuable resource for the advancement of research in the field of infectious disease informatics.
- IDQuAD was created using a diverse collection of recent texts related to infectious diseases sourced from multiple outlets, combined with LLM. Creative methodologies, such as question generation from answer and counterfactual reasoning, were employed in the construction of this dataset.
- To explore the effectiveness of IDQuAD, this study fine-tuned a variety of LLMs to evaluate their performance on QA tasks. Furthermore, the study compared the performance of these models on infectious disease QA tasks in different scenarios, including zero-shot, few-shot, and Chain-of-Thought (CoT) [7] prompting approaches.

Through these contributions, this study aims to significantly improve the tools available for modeling infectious diseases, ultimately leading to more effective public health responses and interventions.

## Related works

### Infectious disease dataset

One of the key datasets in infectious disease research is CORD-19 [8], which was released in response to the COVID-19 pandemic. Developed through collaboration between institutions such as the Allen Institute for Artificial Intelligence and Microsoft Research, CORD-19 integrates more than 140,000 scientific papers on COVID-19 and related coronaviruses such as SARS and MERS.

Similarly, the COVID-QA dataset [9], built on the CORD-19 foundation, provides 2,019 question-answer pairs derived from the biomedical literature annotated by experts. Models fine-tuned on COVID-QA have shown better performance compared to those trained on generic datasets, such as SQuAD [10], highlighting the value of domain-specific data in improving accuracy for disease-related inquiries.

However, both CORD-19 and COVID-QA are limited to infectious diseases related to the coronavirus and rely solely on academic papers as their primary data source. This shows the lack of datasets that cover a wide range of infectious diseases and are collected from a variety of sources. Therefore, datasets are needed that cover a wide range of pathogens and integrate multiple data sources to better prepare for future outbreaks.

### Question-answering dataset

The construction of QA datasets has been pivotal in advancing natural language processing (NLP) systems across various domains. In the medical field, domain-specific QA datasets have been developed to effectively handle medical expertise and complex information. For example, MedQuAD [11] is a large medical QA dataset of nearly 47,000 pairs collected from trusted medical websites and organizations such as MedlinePlus and the National Library of Medicine. By providing authoritative high-quality medical information, this data set helps models generate accurate and reliable answers to complex medical questions. This has proven to be a valuable resource for researchers developing artificial intelligence systems to address medical-specific inquiries with precision.

Similarly, PubMedQA [12] addresses the need for reasoning about complex biomedical research, offering well-founded answers based on the scientific literature. Additionally, MEDQA [13] is designed to simulate real-world medical decision-making scenarios, enabling QA models to develop reasoning skills akin to those of medical professionals. Assesses the clinical knowledge and reasoning capabilities required to solve complex medical problems, contributing significantly to the evaluation and enhancement of QA models in clinical settings. Specific information about the medical QA dataset is in Table 1.

**Table 1:** Comparison of medical domain QA datasets.

Feature	PubMedQA	MedQuAD	MEDQA	COVID-QA
Size	211k QA pairs	47.5k QA pairs	61.1k questions	2.0k QA pairs
Source	PubMed abstracts	NIH websites	USMLE, MCMLE	CORD-19 articles
Year	2019	2018	2020	2020
Answer Type	Subjective	Subjective	Objective	Subjective

Table notes: Comparison of major medical domain QA datasets, highlighting differences in size, data sources, publication year, and answer type.

Despite these advances, the field of infectious disease lacks dedicated QA datasets, which limits the ability to provide accurate and reliable information in this critical area of healthcare. This can make it difficult for a rapid and informed public health response in the event of an outbreak of infectious disease. To overcome these limitations, our study aims to build a comprehensive QA dataset covering various infectious diseases. To this end, we developed a natural, consistent, and dedicated infectious disease-specific dataset based on the SQuAD format, a representative QA dataset. Our proposed dataset provides researchers with structured and reliable information to improve the performance of NLP models and enables QA systems to provide accurate and up-to-date answers for various infectious diseases.

## IDQuAD

### Data collection

As a first step in data collection, we selected 104 infectious diseases from the New South Wales (NSW) Government’s ‘A-Z of Infectious Diseases’ website. Using these disease names, we searched PubMed and arXiv to collect medical papers. The PubMed data was searched using the Dimensions AI platform, focusing on open access (OA) articles from journals indexed in PubMed, while the arXiv API was used to automatically collect metadata such as title, author, abstract, publication date, and DOI. Duplicates between sources were removed to ensure the integrity of the dataset, resulting in a total of 43,231 medical papers.

The news data was collected by crawling the World Health Organization’s Disease Outbreak News (DON). This process yielded 3,131 news articles relevant to the selected infectious diseases. Furthermore, patent data were sourced from the Pandemic Prevention Big Data Exchange, contributing 48,364 patent documents to the dataset. The metadata collected was stored in an Excel file to serve as a basis for the subsequent data processing pipeline, and the abstracts of the metadata were used in the study.

All data collection procedures complied with the terms and conditions of each data source:

- *PubMed*: We restricted contexts to open-access(OA) content and complied with the reuse terms of the respective article licenses. We do not redistribute third-party full texts.
- *arXiv*: Metadata and abstracts were accessed via the official API in line with arXiv’s policies and attribution requirements.
- *WHO Disease Outbreak News*: We used publicly posted reports for non-commercial research without altering the original meaning.
- *Pandemic Prevention Big Data Exchange*: Used under the portal’s terms of service. We do not redistribute full patent documents.

After data collection, metadata from various sources was integrated and pre-processed to build a refined dataset. First, the archive data were crawled and metadata items such as Title, Year, and Abstract were extracted and stored in JSON file format, which was then saved in an Excel file. The dataset was filtered to include data only from the years 2000 to 2024.

For PubMed data, metadata for each infectious disease was collected through the Dimensions AI platform under the following conditions: publication year 2000–2024, Journal List: PubMed, and Open Access: All OA. Titles, abstracts, and infectious disease names were extracted and saved in an Excel file. Similarly, for data from the Pandemic Prevention Big Data Exchange, titles, years, and abstracts were extracted, limiting the data to the period 2000-2020. Duplicates across the three sources were removed, and cleaned data was merged into a single Excel file.

The pre-processing steps included the removal of unnecessary text, such as symbols, to retain only relevant information. The final dataset was organized into columns labeled as follows: disease name, title, abstract, and year. News and patent data were also preprocessed in a similar way, selecting data from 2000 to 2024 and saving the output as an Excel file containing the disease name, year and content.

To generate QA pairs, we first develop a context *C* by pre-processing sources such as abstracts of research papers, patent documents, and news articles. Patent documents are reformulated in the third person, whereas the abstracts and news articles are condensed into 3-5 sentences. For each *C*, a single QA pair is generated by initially formulating an answer *A* and subsequently deriving a corresponding question *Q* based on *A*. This methodology ensures the consistency of the QA pairs by generating the question from the answer [14]. Furthermore, it mitigates ambiguities and inaccuracies that may arise from generating the question prior to the answer. Consequently, the question is intricately linked to the most pertinent information within the text, thus enhancing the overall quality and relevance of the QA pairs. The answer *A* generated answer serves as the ground truth for constructing the final QA pair. The volume was too large to generate QA pairs from all source data, so we randomly selected a small subset per source to form the final QA pairs.

**Algorithm 1** Answer Regeneration via Counterfactual Simulation

**Input:** ⟨Context C,Question Q⟩ for *i* = 1 to *N*

Number of repetitions M, Threshold T, Response R, Original Answer A, New Answer A′

**Output:** $\langle C,Q,A^{\prime }\rangle$

**for** ⟨C,Q⟩ in Dataset **do**

**Prompt:**

• System message:

Counterfactual Thinking Process

• User message:

generate_answer_prompt(*C*, *Q*)

**for** Repeat to M **do**

R←Llama3 generate()

F1←Compute_F1(R,A)

**if** F1 ≥ T **then**

A′ ← R

Break the loop

**end if**

**end for**

**end for**

### Question & answer generation

**Fig 2:**
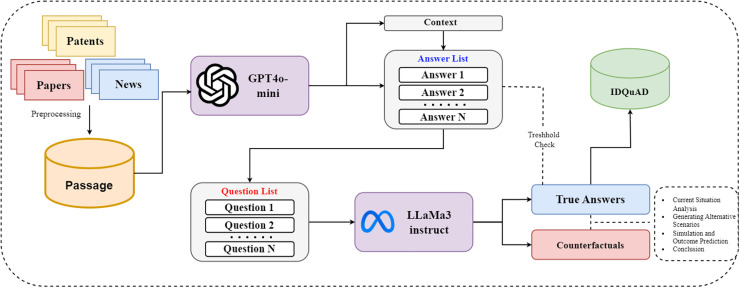
Framework for generating the IDQuAD. This framework generates the IDQuAD by preprocessing data from papers, patents, and news, then using GPT4o-mini for answers and LLaMa3-instruct for questions. A threshold check ensures quality, with counterfactual reasoning applied for refinement.

Our objective is to generate high quality QA pairs to effectively train language models for tasks related to infectious disease. Specifically, our framework compares two primary stages: (1) question-answer generation and (2) answer generation. Fig 2 delineates the comprehensive framework for constructing the QA dataset.

In the initial phase-QA generation- we employ the GPT-4o-mini. GPT-4o-mini is a streamlined variant of the GPT-4 architecture engineered for optimized text generation efficiency while maintaining a robust balance between output quality and computational resource utilization.

### Answer regeneration

The answer *A* can lack causality with respect to the question *Q* and the context *C*. Therefore, we generate an answer *A’* to *Q* using counterfactual simulation. We use Llama3-8B-Instruct [15] and employ CoT prompting to guide the model in following the counterfactual simulation process to generate *A’*. Llama3-8B-Instruct contains 8 billion parameters and has been optimized to perform a variety of tasks through instruction-based learning. Specifically, it has undergone instruction tuning for MMLU, GPQA, HumanEval, GSM, and MATH, which gives it strengths in complex reasoning and problem solving.

Causality in this context refers to the logical relationship between cause and effect, which is essential to ensure that the generated QA pairs are contextually grounded and relevant. By addressing causality, we aim to produce QA pairs that better reflect the complexity of reasoning required in infectious disease scenarios, where understanding causal links can directly impact the quality of insights derived from the data.

Counterfactual simulations embody one of the key aspects of human intelligence: counterfactual thinking. This process involves evaluating alternative outcomes to a given scenario and drawing conclusions based on explicit causal relationships. For example, scenarios such as ‘how might the pandemic have evolved differently if virus containment measures had been implemented earlier’ allow the model to explore diverse possibilities. Previous research has used counterfactual data to train models’ causal representation [16]. Building on this, we leverage CoT prompts to actively guide the model through counterfactual thinking during the QA generation process. Details about the CoT prompting design and the counterfactual thinking process are provided in Appendix C. By doing so, the model can simulate scenarios more effectively, resulting in improved causal inferences.

To balance quality and coverage of the generated QA pairs, we set the F1-score threshold to 0.7. A higher threshold could exclude valid pairs due to minor lexical variations, whereas a lower threshold might allow low-quality pairs. This value is widely adopted in QA dataset studies, as detailed in Sect 3.B. Following MEDQA [13], we randomly sampled 1,000 QA pairs(18.4%) from 5,439 pairs. Experts from the Pandemic Prevention Big Data Exchange assessed each pair (i) whether each question can be answered by the evidence from the context and (ii) the relevance of the pairs. Through this systematic approach, the IDQuAD dataset was finalized, achieving a high standard of relevance and reliability.

### Characteristics

IDQuAD is a comprehensive dataset of 5,439 QA pairs (English-only) carefully curated to support the training of LLMs in the field of QA of infectious diseases. A representative QA sample is shown in Fig 1. Each QA pair within the dataset is structured with several essential components to ensure robustness and effectiveness in model training. These components include:

- Question: Designed to evaluate the model’s ability to reason and extract relevant information related to infectious diseases.
- Answer: Provides an accurate and concise answer to the question posed.
- Context: Provides the background information necessary to derive the correct answer, ensuring that the model can understand and effectively use relevant data.
- ID: A unique identifier assigned to each QA pair, to facilitate efficient categorization, management, and retrieval within the dataset.
- Disease Name: Metadata indicating the specific infectious disease associated with the QA pair, allowing for organized classification and targeted searches based on disease categories.

The variety of infectious diseases covered in IDQuAD is extensive, encompassing dozens of different diseases to ensure comprehensive coverage and applicability in various scenarios. Despite this wide-ranging inclusion, a subset of diseases is more frequently represented within the dataset. In particular, COVID-19 appears approximately twice as often as other infectious diseases, reflecting its significant global impact and the increased research interest surrounding it.

**Fig 3:**
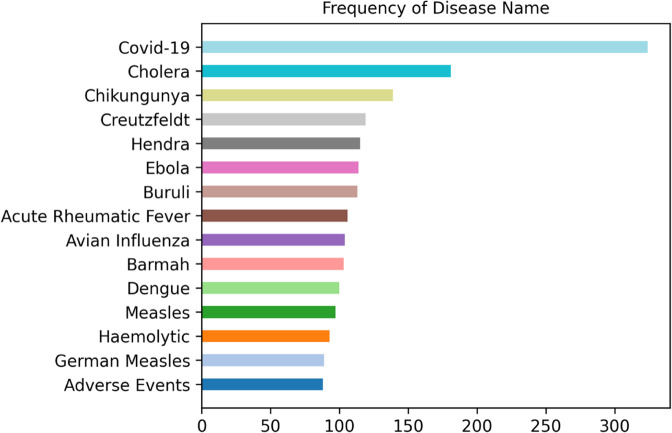
Frequency of disease name within IDQuAD. “Hemolytic” refers to hemolysis-related infectious diseases, while “Adverse Events” are associated with vaccine-related adverse effects.

Fig 3 shows the frequency of infectious diseases within the IDQuAD dataset. It provides a clear perspective of the emphasis placed on various infectious diseases. This visualization helps researchers understand the composition of the dataset and the relative importance of different diseases within the training material.

Overall, the structured composition of IDQuAD, with its well-defined components - Question, Answer, Context, ID, and Disease Name - ensures that the dataset is both comprehensive and highly organized.

## Experiments

This section outlines two main experiments conducted to evaluate the performance of LLMs using IDQuAD. The objective is to assess the effectiveness of fine-tuning and to evaluate the models’ generalization capabilities through zero-shot and few-shot learning, with and without Chain of Thought prompting. The experiments focus on key metrics such as Exact Match (EM) and F1 score to determine the models’ ability to handle infectious disease-specific question-answer tasks.

### Fine-tuning LLM with IDQuAD

In the first experiment, we evaluate the impact of fine-tuning LLMs with IDQuAD on QA performance. We chose the Mistral-7B [17] model, a general-purpose LLM, as the model to evaluate. We test Mistral-7B before and after fine-tuning to determine the impact of the IDQuAD dataset on performance in infectious disease-related QA tasks. In the 1-shot and 5-shot settings, the models are provided with one and five examples respectively, simulating scenarios where only limited labeled data is available.

EM(p,g)=⊮(p=g) (1)

$$F1=\frac{2\times precision\times recall}{precision+recall}$$ (2)

Specifically, the model first undergoes a baseline evaluation on IDQuAD dataset without any fine-tuning to determine its performance. In this step, the EM and F1 scores, the evaluation metrics of the QA task, are measured to assess how accurately the model answers the questions. The model is then fine-tuned on the IDQuAD dataset. After fine-tuning, the model is reevaluated on the IDQuAD dataset under the same few-shot setting, and its EM and F1 scores are compared with the results before fine-tuning. Equation (1) is the calculation expression for the EM, and equation (2) is the calculation expression for the F1 score.

### Comparison of LLM performance with IDQuAD

**Fig 4:**
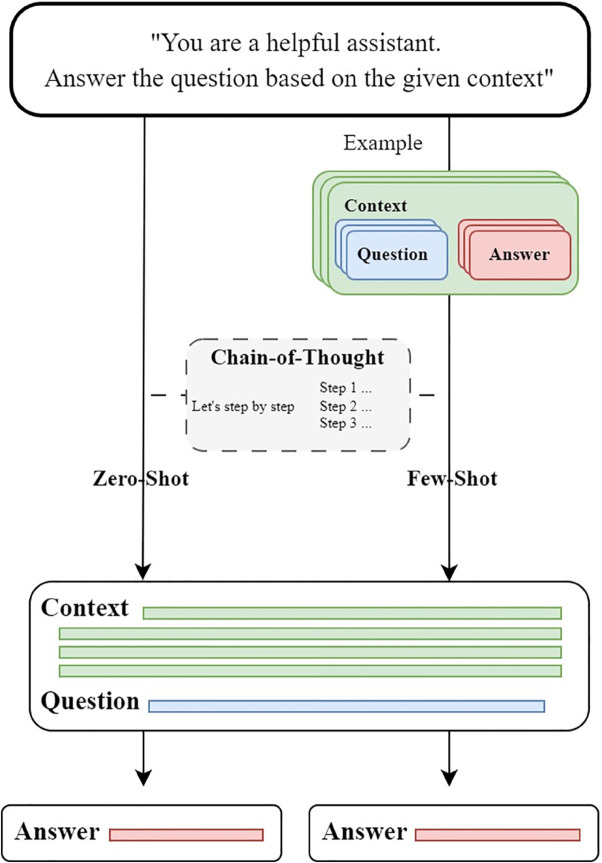
Experiment pipeline for evaluating model performance on IDQuAD using zero-shot, few-shot, and chain-of-thought prompting.

The second experiment evaluates the models’ performance on the IDQuAD dataset across zero-shot, 1-shot, and 5-shot learning setups, comparing EM and F1-scores under each condition. This experiment aims to test the ability of the models to generalize to the infectious disease QA task with varying levels of task-specific exposure. The experiment pipeline is shown in Fig 4. The models tested in this experiment are GPT-3.5-turbo, GPT-4o-mini, Llama3-8B-instruct [15], Mistral-7B, Gemma2-9B-instruct [18], MedAlpaca [5], and a fine-tuned version of Mistral. For each model, we conducted three rounds of evaluation in zero-shot, 1-shot, and 5-shot settings. The average EM and F1 scores are calculated from these three runs. In the zero-shot setting, the models answered questions without prior task-specific examples.

**Fig 5:**
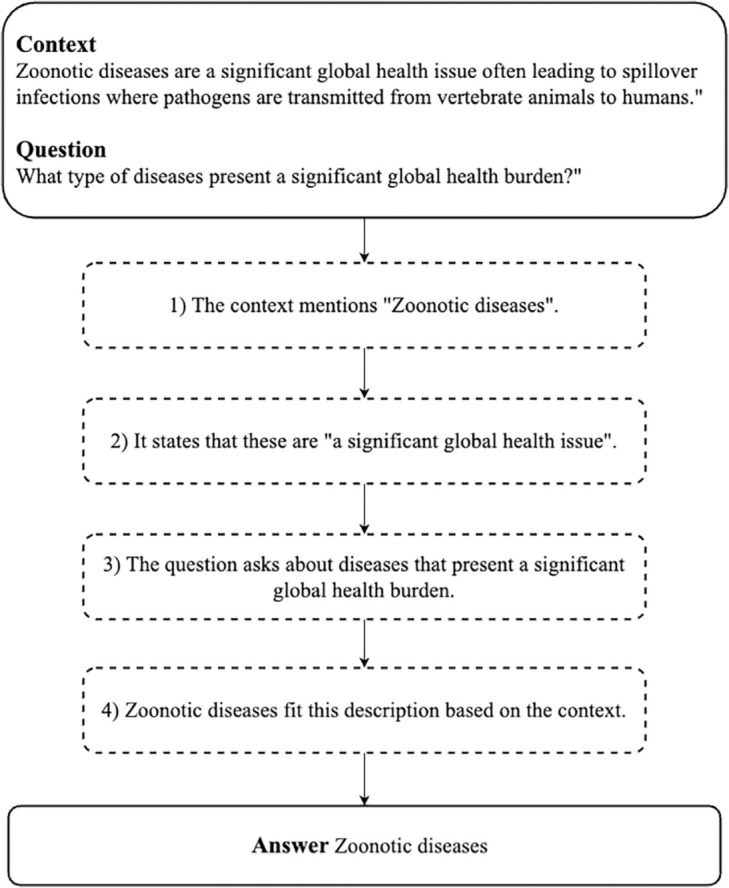
CoT prompt reasoning process.

To further investigate the reasoning abilities of the models, we also use CoT prompting. This technique encourages models to generate step-by-step reasoning when answering complex questions. Fig 5 illustrates an example of CoT prompt reasoning process in our experiment. The CoT prompting design encourages the model to engage in counterfactual thinking by structuring queries to explore hypothetical scenarios. This approach enhances the model’s reasoning ability in infectious disease-related QA tasks.

We compared CoT-prompted results with those generated without CoT to determine if reasoning-based techniques improve performance in infectious disease QA scenarios. However, Llama3-8B-Instruct and MedAlpaca models were not subjected to CoT prompting due to the extended inference time required for such operations.

### Experiment setup

Table 2 shows our experimental setup. In the experiment, we fine-tuned the MistralAI Mistral-7B-Instruct-v0.3 model. The GPU environment used was NVIDIA L40S * 8, providing high computational efficiency. The learning rate was set to 2e-5, with a maximum sequence length of 512 tokens. Training was carried out over 5 epochs using the AdamW optimizer in PyTorch. Gradient clipping was applied to stabilize training and prevent exploding gradients, while a weight decay of 0.01 was used to minimize overfitting.

**Table 2:** Experimental setup.

Feature	Value
Model	MistralAI/Mistral-7B-Instruct-v0.3
GPU Environment	L40S × 8
Learning Rate	2e-5
Max Sequence Length	512
Number of Epochs	5
Optimizer	AdamW

Table notes: Details of the experimental setup, including model, GPU environment, and optimization parameters.

## Results and discussion

The results of our experiments provide valuable insight into the performance of general-purpose and domain-specific LLMs on the IDQuAD dataset, both before and after fine-tuning, and under zero-shot, few-shot, and CoT prompt conditions. We focus on two main metrics: Exact Match and F1 score, which provide a comprehensive view of the models’ abilities to handle infectious disease-specific QA tasks.

### Fine-tuning performance

**Fig 6:**
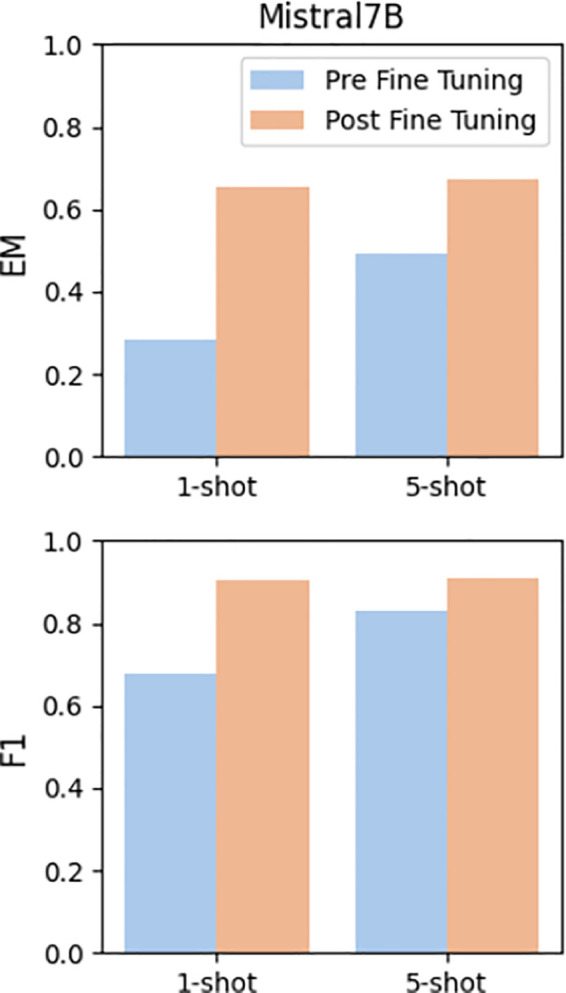
Comparison of mistral-7B performance. Pre and post fine-tuning on IDQuAD dataset (EM and F1 scores).

The results before pre and post fine-tuning are shown in Fig 6. The results show a significant improvement in the performance of Mistral after fine-tuning with IDQuAD. The EM score, which measures the percentage of questions where the exact answer is produced, showed a significant increase. In the 1-shot setting, the EM score improved from 0.28 before fine-tuning to 0.65 after fine-tuning. Similarly, in the 5-shot setting, the model’s EM score increased from 0.50 to 0.67 post-fine-tuning. This improvement clearly indicates that fine-tuning with domain-specific data enables the model to perform better in providing exact answers to infectious disease-related questions.

The F1 score, which balances precision and recall, also showed significant improvements. Before fine-tuning, the F1 score for the 1-shot setting was 0.49, which increased to 0.60 post-fine-tuning. In the 5-shot setting, the pre-fine-tuning F1 score of 0.83 slightly improved to 0.91 after fine-tuning. This suggests that while the model was relatively strong at retrieving relevant information before fine-tuning, its ability to recall specific information improved further after being fine-tuned with IDQuAD.

### Comparison of LLM performance with IDQuAD

The second experiment evaluated the generalization capability of the models on the IDQuAD dataset in zero-shot, 1-shot, and 5-shot settings, with and without CoT prompts. The experiment result is shown in Table 3. In the zero-shot setting, the fine-tuned Mistral-7B model achieved the highest scores with 42.37% EM and 83.30% F1 without CoT. However, CoT prompts reduced both metrics. This performance degradation is due to three interacting factors:

- Format-metric mismatch: IDQuAD’s answers are typically short (e.g., entity), while CoT encourages multi-sentence rationales. When calculating EM ans F1 based on tokens, the longer the output, the less accurate it is, and exact string matching will not occur, which will lower the score even if the correct span appears.
- Instructional drift due to counterfactual template: Our CoT template emphasizes counterfactual exploration (Appendix C). When in-context examples do not follow the template, zero-shot CoT could introduce hypothetical statements and reservations that are not grounded in the context provided, increasing lexical variability and off-target content.
- Lack of answer style calibration: With no task-specific examples, the model is not calibrated to end with a concise final answer. If 1 or 5 demonstrations are provided, CoT becomes helpful—reasoning remains but the exemplars anchor the required answer format. It explains the performance improvement we observe in the 1-shot and 5-shot settings.

**Table 3:** Experimental results of different models on IDQuAD dataset in zero-shot, 1-shot, and 5-shot settings with and without CoT prompting.

Model	IDQuAD (zero-shot)		IDQuAD (1-shot)		IDQuAD (5-shot)	
	EM	F1	EM	F1	EM	F1
Llama3-8b-instruct	-	15.62	9.92	-	10.67	-
Medalpaca	9.40	49.08	20.10	60.09	40.20	68.46
**w/o CoT**						
GPT-3.5-turbo	1.43	38.10	29.43	62.29	34.83	67.57
GPT-4o-mini	-	36.88	27.89	63.65	41.13	73.17
Gemma2-9b-instruct	27.00	55.38	35.90	51.02	56.60	69.13
Mistral-7b	3.00	48.37	28.50	67.90	49.10	83.08
Mistral-7b Fine-tuning (ours)	**42.37**	**83.30**	65.47	90.28	67.30	91.02
**w/ CoT**						
GPT-3.5-turbo	-	33.21	49.33	81.63	51.17	83.34
GPT-4o-mini	-	15.18	46.67	81.31	50.10	83.70
Gemma2-9b-instruct	4.20	36.83	60.20	86.50	60.20	87.92
Mistral-7b	0.10	26.36	45.30	80.64	51.70	83.26
Mistral-7b Fine-tuning (ours)	0.30	30.69	**71.87**	**93.05**	**69.40**	**91.61**

Table notes: The table presents experimental results on the IDQuAD dataset. Underlined numbers are second-best performances, and bold numbers indicate the best performance. If a value was too small or zero, it is represented as ‘-’. All numbers are percentages.

All models showed significant improvements in both 1-shot and 5-shot settings. The fine-tuned Mistral-7B model again outperformed the others, achieving 65.47% EM and 90.28% F1 in the 1-shot setting, and 69.40% EM and 91.61% F1 in the 5-shot setting. This confirms that even limited examples can significantly improve the performance of the model. GPT-3.5-turbo and GPT-4o-mini, which initially underperformed in zero-shot, showed drastic improvement with CoT in the 1-shot and 5-shot scenarios. The results highlight the importance of fine-tuning general-purpose models on domain-specific datasets such as IDQuAD to improve performance in QA tasks. While CoT prompting proved effective in the few-shot setting, it did not provide the same benefits in the zero-shot scenario. Fine-tuning coupled with CoT produced the highest scores in 1-shot and 5-shot settings, confirming the utility of task-specific examples and reasoning-based techniques for infectious disease QA tasks.

### Discussion

Overall, fine-tuning general-purpose models with IDQuAD not only improved their ability to handle infectious disease-related queries but also demonstrated that specialized datasets are key to improving LLM performance in niche domains. This was particularly evident in the significant performance gains observed with Mistral-7B after fine-tuning, where the model showed improvements in Exact Match (EM) and F1 scores across various shot settings. In addition, the fine-tuned version of Mistral-7B without CoT prompting performed better in zero-shot scenarios compared to other models with CoT, suggesting that fine-tuning with a domain-specific dataset can sometimes compensate for the lack of step-by-step reasoning prompts in simpler tasks.

## Conclusion

In this study, we introduced IDQuAD, a novel and comprehensive dataset specifically designed for infectious disease-related question-answering tasks. IDQuAD was constructed through a meticulous process of data collection from various sources, including medical papers, news, and patents. The dataset is enriched with high-quality QA pairs generated using advanced LLM and counterfactual reasoning techniques to ensure relevance and accuracy in answering infectious disease queries. Through the development and fine-tuning of LLMs on this domain-specific dataset, we demonstrated significant improvements in performance across both zero-shot and few-shot settings. The fine-tuned Mistral-7B model showcased marked gains in EM and F1 scores, confirming the utility of domain-specific data in enhancing the accuracy and relevance of LLMs for infectious disease queries. Additionally, while the inclusion of CoT prompting proved useful in few-shot settings, its utility in zero-shot tasks was less pronounced, suggesting that a careful balance between domain-specific fine-tuning and task-specific techniques is necessary.

### Limitations

Despite the promising results, our study has several limitations. Our focus on a single prompting strategy limits the exploration of other prompt engineering methods that could potentially enhance complex reasoning tasks. Furthermore, our methodology relies on commercial APIs for dataset generation, which makes the process dependent on external services and subject to potential cost and availability changes. To mitigate this, future work could adopt a more automated data extraction system, such as those that automate the extraction of data from large repositories [19].

Our fine-tuned model uses a traditional language model architecture. The performance could be enhanced by exploring more sophisticated approaches, as demonstrated in studies that use non-textual data representations or advanced deep learning models to achieve higher accuracy [20].

### Future work

To address these limitations and further improve our system’s performance and scalability, we propose several future work directions. To accelerate the dataset creation process, we will explore methods to parallelize the data generation workflow, inspired by automated data extraction concepts [19]. This would significantly reduce the time needed to build large-scale datasets.

To enhance model performance and training efficiency, we will investigate various parallelization strategies for model training, such as data or model parallelism. We also plan to explore a broader range of pre-trained models and transfer learning strategies, as shown in studies that use models for classification tasks [21].

Finally, future research will explore applying the IDQuAD dataset to other NLP tasks beyond QA, such as text classification and summarization, to broaden its utility for infectious disease research.

## Supporting information

S1 Fig Prompt for counterfactual simulation. S1 Fig illustrates a 4-step process for designing counterfactual simulation prompts, helping the model analyze scenarios by evaluating the current situation, generating alternative possibilities, predicting outcomes, and drawing conclusions based on causal relationships.
(TIFF)

S2 Fig User prompt. S2 Fig guides the model to provide short and accurate answers to questions based on a given context. The structure ensures that answers strictly adhere to the information present in the context, avoiding any inference or expansion beyond what is explicitly stated.
(TIFF)

S1 File IDQUAD dataset. (JSON)

S2 File The main code for fine-tuning the Mistral model. (PY)

S3 File The code used to create the dataset. (PY)
