# Supplementary figures and images for: IDQuAD: Infectious disease question and answering dataset
Source: PLoS One. 2025 Oct 9;20(10):e0333075. doi: 10.1371/journal.pone.0333075 (PMC12510504; doi:10.1371/journal.pone.0333075)

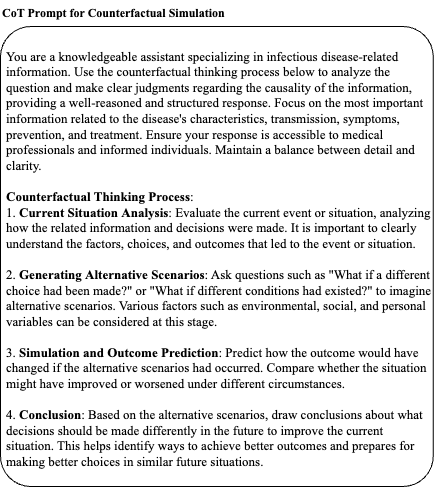

Supplement: S1 Fig — illustrates a 4-step process for designing counterfactual simulation prompts, helping the model analyze scenarios by evaluating the current situation, generating alternative possibilities, predicting outcomes, and drawing conclusions based on causal relationships. (TIFF) [file pone.0333075.s001.tif]

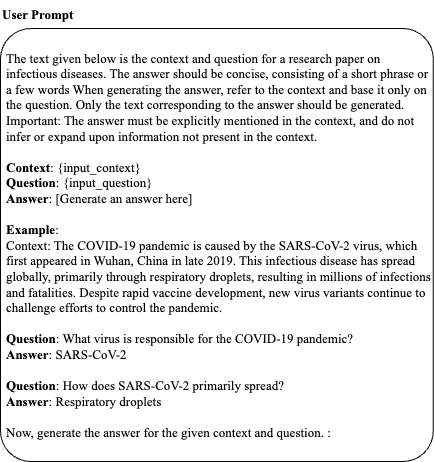

Supplement: S2 Fig — guides the model to provide short and accurate answers to questions based on a given context. The structure ensures that answers strictly adhere to the information present in the context, avoiding any inference or expansion beyond what is explicitly stated. (TIFF) [file pone.0333075.s002.tif]
